# What Matters Most for Predicting Survival? A Multinational Population-Based Cohort Study

**DOI:** 10.1371/journal.pone.0159273

**Published:** 2016-07-19

**Authors:** Noreen Goldman, Dana A Glei, Maxine Weinstein

**Affiliations:** 1 Office of Population Research, Princeton University, Princeton, NJ, United States of America; 2 Center for Population and Health, Georgetown University, Washington, DC, United States of America; 3 Center for Population and Health, Georgetown University, Washington, DC, United States of America; Geisel School of Medicine at Dartmouth College, UNITED STATES

## Abstract

Despite myriad efforts among social scientists, epidemiologists, and clinicians to identify variables with strong linkages to mortality, few researchers have evaluated statistically the relative strength of a comprehensive set of predictors of survival. Here, we determine the strongest predictors of five-year mortality in four national, prospective studies of older adults. We analyze nationally representative surveys of older adults in four countries with similar levels of life expectancy: England (*n* = 6113, ages 52+), the US (*n* = 2023, ages 50+), Costa Rica (*n* = 2694, ages 60+), and Taiwan (*n* = 1032, ages 53+). Each survey includes a broad set of demographic, social, health, and biological variables that have been shown previously to predict mortality. We rank 57 predictors, 25 of which are available in all four countries, net of age and sex. We use the area under the receiver operating characteristic curve and assess robustness with additional discrimination measures. We demonstrate consistent findings across four countries with different cultural traditions, levels of economic development, and epidemiological transitions. Self-reported measures of instrumental activities of daily living limitations, mobility limitations, and overall self-assessed health are among the top predictors in all four samples. C-reactive protein, additional inflammatory markers, homocysteine, serum albumin, three performance assessments (gait speed, grip strength, and chair stands), and exercise frequency also discriminate well between decedents and survivors when these measures are available. We identify several promising candidates that could improve mortality prediction for both population-based and clinical populations. Better prognostic tools are likely to provide researchers with new insights into the behavioral and biological pathways that underlie social stratification in health and may allow physicians to have more informed discussions with patients about end-of-life treatment and priorities.

## Introduction

“*The impetus to foretell death is both fundamental and ancient*”

(p. 23) [1]

Laymen, social scientists, and health researchers have long shared a fascination with predicting a person’s longevity or future age at death. In a modern day version of fortune telling, the internet is replete with websites and “apps,” with such evocative names as “Death Clock,” “Death Timer,” and “Deadline,” designed to predict age at death or time remaining before death on the basis of a battery of simple questions.

Clinicians have developed more scientifically based prognostic tools to predict how long a person will survive, most often a patient who is severely ill. Such estimates help physicians assess the costs and benefits of interventions and assist patients and families in making healthcare decisions. Nevertheless, clinicians often avoid making prognoses—not only because of the unpleasantness, anxiety, and sadness that they experience when communicating such information to patients, but also because they are aware of the enormous uncertainty inherent in their predictions [1].

Social scientists and epidemiologists have largely relied on statistical models to predict mortality from a diverse set of information. Based on these models, researchers have identified variables that show strong associations with the probability of dying in a follow-up period, typically without making explicit forecasts. The explanatory variables in these models comprise both proximate determinants of survival (e.g., disease and disability) and more distant demographic, social, economic, and environmental factors. Information about these predictors is typically obtained from responses to questions in household surveys of middle-aged and older adults, with follow-up data on survival generally derived from registration systems (see, for example, [2–4]).

The criteria used to develop these statistical models and prognostic tools differ substantially across researchers. One explanation for this variability is disciplinary myopia: scholars often focus on measures most prominent in their discipline, thereby failing to consider the relative importance of a comprehensive set of predictors. It is also the case that, until the recent proliferation of biosocial surveys [5, 6], very few datasets incorporated a sufficiently broad set of demographic, social, health, and clinical variables along with linkages to subsequent mortality. In this paper, we use the extensive information collected in four recent biosocial surveys—in England, the US, Costa Rica and Taiwan—each of which is based on a large, national sample. We identify the best predictors of survival and assess robustness of these findings with multiple measures of discrimination. We address two specific questions. First, what factors matter most in determining whether an older adult will die in the next five years? Second, are the principal predictors the same across countries with similar levels of life expectancy but distinct social environments, levels of affluence, and access to health care?

## Materials and Methods

### Data

We use a cohort study design based on data from four biosocial surveys fielded around 2005. Each is based on a nationally representative sample of older adults: the 2005–2006 wave of the National Health and Nutrition Examination Survey in the US (NHANES); the 2006 wave of the Social Environment and Biomarkers of Aging Study in Taiwan (SEBAS); the 2004–2006 wave of the Costa Rican Study on Longevity and Healthy Aging (CRELES); and the 2004–2005 wave of the English Longitudinal Study of Aging (ELSA).

The main analysis is restricted to older respondents who provided a blood sample and for whom vital status could be verified: NHANES (*n* = 2023, ages 50+); SEBAS (*n* = 1032, ages 53+); CRELES (*n* = 2694, ages 60+); and ELSA (*n* = 6113, ages 52+). As a sensitivity check, we repeat the analysis on the full samples of interviewed respondents (n = 2214 for NHANES; n = 1284 for SEBAS; n = 2827 for CRELES; n = 8559 for ELSA). (See S1 Table, which provides details regarding sampling design and response rates for the interviews and biomarker collections for each dataset.).

### Ethics Statement

All four surveys obtained written, informed consent from all participants and received human subjects approval from the institutional review boards (IRB) at the institutions conducting the studies: the Ethical Science Committee of the University of Costa Rica (VI-763-CEC-23-04) [CRELES]; the Multi-Centre Research Ethics Committees in England [ELSA]; Princeton University IRB (#1848, #2193, #2791, #3391), Georgetown University IRB (#1999–195), and the Joint IRB in Taiwan (NIFP-IRB-2000-01) [SEBAS]; NCHS Research Ethics Review Board (Protocol #98–12) [NHANES].

### Mortality

Administrative records are used to determine respondents’ survival status, and, for CRELES, these records are supplemented with survey follow-up data (see S1 Appendix for more details). Given differences across datasets in the length of mortality follow-up, we restrict our analysis to mortality within five years post-survey. For ELSA, the public-use dataset includes only the year (not the month) of death, and thus, we model death by the end of 2009 (mean follow-up = 5.0 years; min = 4.4, max = 5.5). For the other three datasets, we censor survivors at five years post-survey with the exception of several foreigners in CRELES (*n* = 61, 2.3%) who are censored at the date of last contact. The average follow-up for CRELES is 4.96 years (maximum = 5 years). The mean and maximum follow-up for NHANES and SEBAS are 5 years. The numbers of respondents who died within the follow-up period are 694 in CRELES, 522 in ELSA, 279 in NHANES, and 112 in SEBAS.

### Predictors

Using clinical, community, and broader population-based samples, researchers have identified a diverse set of predictors of survival. These predictors can be classified into two groups: (1) environmental variables—demographic characteristics, socioeconomic status, psychosocial factors (e.g., social integration), and health-related behaviors (e.g., smoking); and (2) measures of underlying health (e.g., biological and clinical measurements associated with chronic disease, self-reported measures of health and disability, and assessments of physical function).

In this analysis, we consider 57 potential predictors—in addition to age and sex—that have been shown previously to predict mortality (see S2, S3 and S4 Tables, which provide details regarding how the predictors are measured). Measures of underlying health comprise three types of variables: (1) biomarkers obtained from physical measurements and blood and urine specimens, (2) self-reported variables, and (3) health assessments, most of which were conducted or provided by the interviewer.

### Analytical Strategy

To minimize selection biases resulting from missing data, we followed standard practices of multiple imputation to handle missing data (see S2 Appendix) [7, 8].

**Fig 1:**
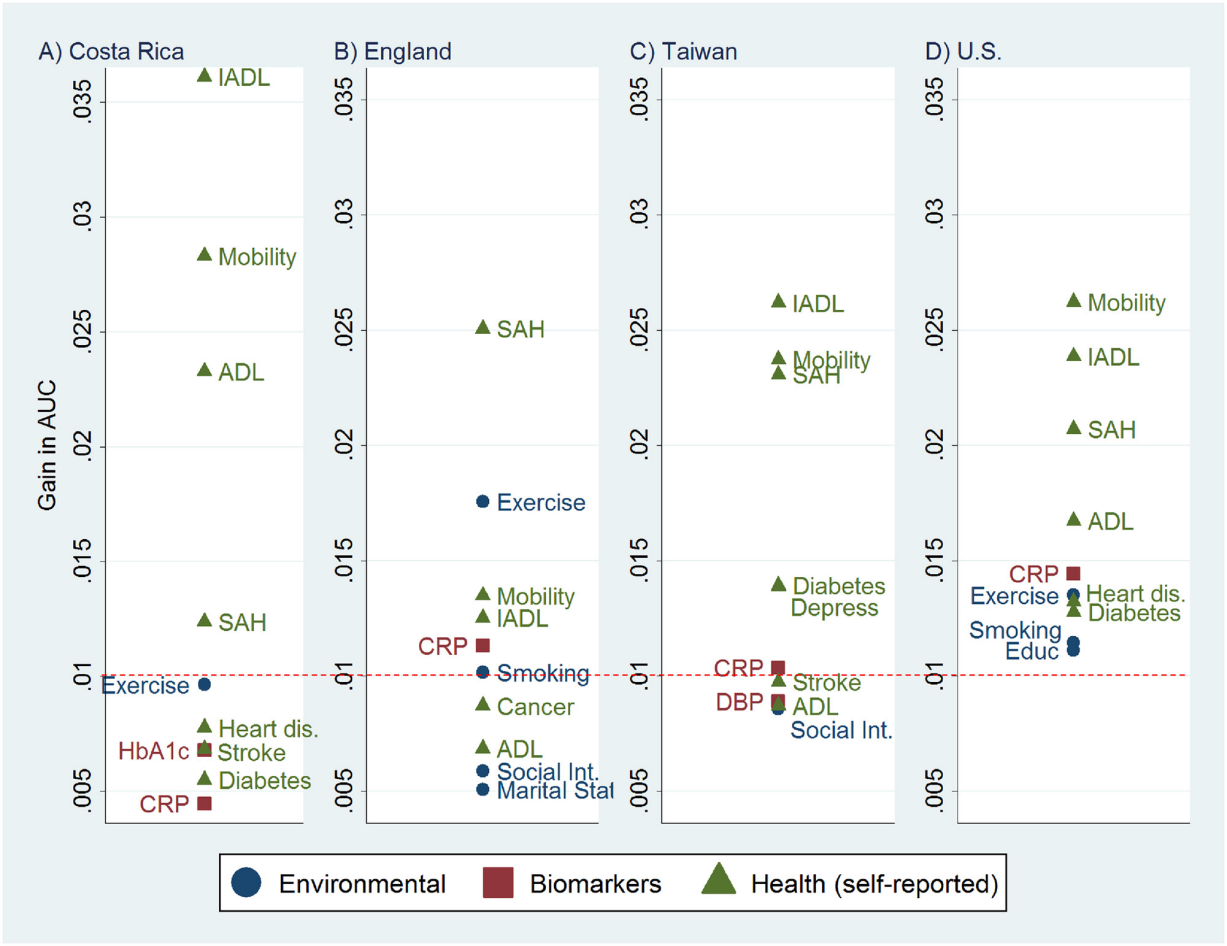
Top Ten Predictors (out of 25 variables available in all four datasets) of Five-Year Mortality After Adjustment for Age and Sex, Ranked by the Gain in AUC, A) Costa Rica, B) England, C) Taiwan, and D) the U.S. Abbreviations: ADL, Activities of daily living; AUC, Area under the receiver operating characteristic curve; CRP, C-reactive protein; Depress, Depressive Symptoms; Educ, Educational attainment; HbA1c, Glycosylated hemoglobin; Heart dis., Heart disease; IADL, Instrumental Activities of Daily Living; SAH, Self-assessed health status, Social Int., Social integration. ^a^ The values for diabetes and depressive symptoms in Taiwan are too close to be distinguishable.

**Fig 2:**
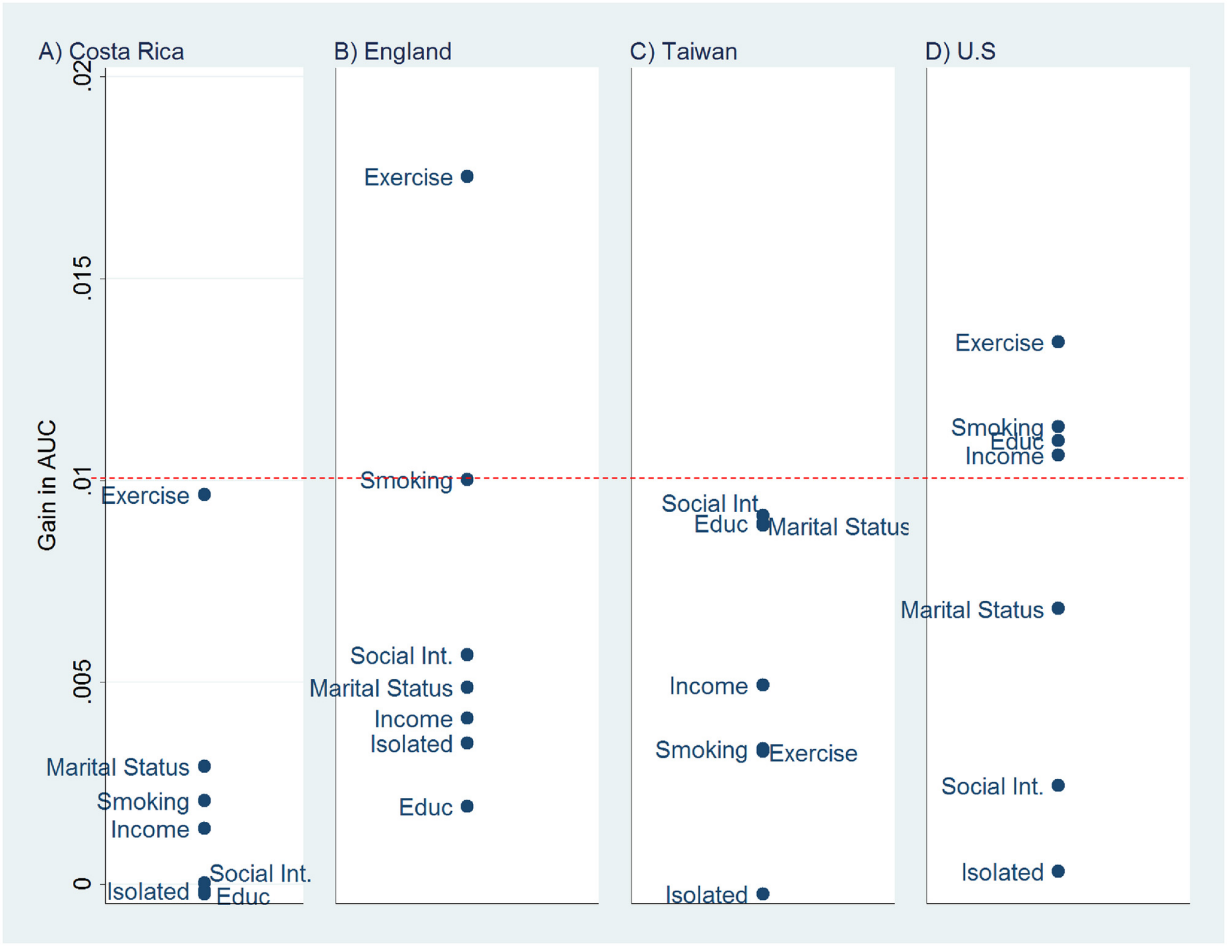
Environmental Predictors of Five-Year Mortality After Adjustment for Age and Sex, Ranked by the Gain in AUC, A) Costa Rica, B) England, C) Taiwan, and D) the U.S. Abbreviations: Educ, Educational attainment; Social Int., Social integration.

**Fig 3:**
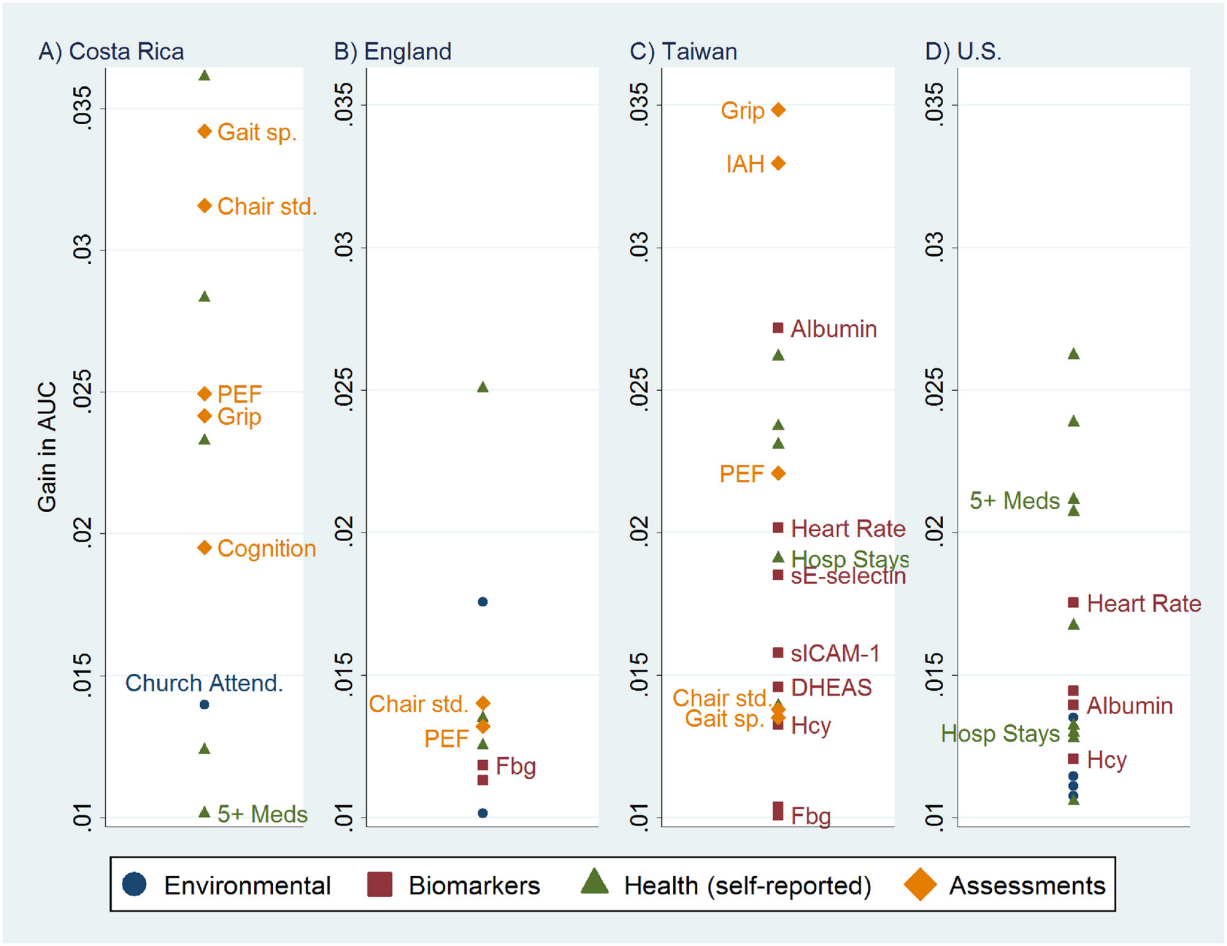
Additional Predictors of Five-Year Mortality that Yield a Gain in AUC≥0.01 After Adjustment for Age and Sex, A) Costa Rica, B) England, C) Taiwan, and D) the U.S. The unlabeled markers represent variables displayed in Fig 1. Abbreviations: 5+ Meds, 5+ Medications; AUC, Area under the receiver operating characteristic curve; Chair std., Chair stand speed; Church Attend., Church attendance; Fbg, Fibrinogen; Gait sp., Gait Speed; Grip, Grip strength; Hcy, Homocysteine; Hosp stays, Number of hospitalizations; IAH, Interviewer-assessed health status; IL-6, Interleukin-6; PEF, Peak expiratory flow; sE-selectin, Soluble E-selectin; sICAM-1, Soluble intercellular adhesion molecule 1.

To account for differential response rates and oversampling, we weighted descriptive statistics (S5 Table). For ELSA, given limited information regarding the timing of deaths, we used a logit model (i.e., with death by the end of 2009 as the outcome). For NHANES, SEBAS, and CRELES, we estimated a Cox hazard model using age as the “clock” to predict age-specific death rates for the five-year period post-survey, thereby allowing the underlying baseline hazard over age to assume whatever functional form best fits the data. All models adjusted for age and sex and were fit separately by country using unweighted data. The use of sampling weights in multivariate regression analysis remains a matter of debate among statisticians (see, for example, [9]). For the rankings presented here, we added each of the remaining predictors one at a time to this model. In the first stage of the analysis (Figs 1 & 2), we examined only variables available for all four surveys; in the second stage (Fig 3), we considered additional variables collected in only some of the surveys.

We tested interaction terms between each predictor and age (linear) to allow for variation in the effect of the predictor across age (i.e., non-proportional hazards). If *p*<0.05 (two-tailed test), we included the interaction along with the main effect for that predictor (see S6 Table, which lists the predictors with non-proportional hazards).

We present the hazard ratios (odds ratios for ELSA) for the top 10 predictors in each country in S7 Table. However, because our objective is to assess the predictive ability of variables rather than the magnitude of the associations, we rely on measures of discrimination to determine the rankings of predictors. Results are based on the most frequently used discrimination measure, the area under the receiver operating characteristic curve (AUC). The receiver operating characteristic (ROC) curve is a graph of sensitivity (i.e., true positive rate) against 1-specificity (false positive rate), calculated for each possible cutoff point for distinguishing between two groups of individuals (e.g., alive or dead). The area under that curve summarizes how well the model discriminates between decedents and survivors. Although there is no scientific basis for evaluating the magnitude of the AUC, Pencina et al. [10] suggest that increases of at least 0.01 denote meaningful improvements. We identify this threshold of 0.01 in our graphical results (Figs 1 & 2).

To assess the robustness of our conclusions, we present the rankings of top predictors based on the AUC as well as on two additional measures of discrimination: a category-free version of the Net Reclassification Improvement (NRI(>0)) and the Integrated Discrimination Improvement (IDI) (see S1–S4 Figs, which show the top ten predictors ranked by the AUC, NRI(>0), and IDI) [10, 11]. See S3 Appendix, which describes how the measures of discrimination are calculated.

## Results

To assess the improvement in the AUC, all models are compared with a basic model—one that uses age as the time metric and controls only for sex. The AUC values for this model are 0.766 for Costa Rica, 0.776 for Taiwan, 0.788 for the U.S., and 0.808 for England.

### Predictors Available in All Datasets

In the first stage of the analysis, we consider 25 predictors that are available for all four countries. Among these 25 variables, self-reported measures of health status dominate the top 10 predictors (Fig 1). The number of instrumental activities of daily living (IADL) limitations, the number of mobility limitations, and the simple measure of self-assessed health (SAH) (i.e., a rating of overall health based on five categories) are among the top four predictors in all countries. One biomarker, C-reactive protein (CRP), is among the top 10 predictors in all four countries. Among the eight other biomarkers, only two appear among the top 10: glycosylated hemoglobin in Costa Rica and diastolic blood pressure in Taiwan. Self-reports of diagnosed heart disease, stroke, diabetes, and cancer appear on the list for some countries, but only in Taiwan does one of these (diabetes) appear among the five strongest predictors.

In Fig 2, we exclude the measures of underlying health from the diagram and consider only the seven environmental predictors, which are likely to exert their influence on mortality via the health measures. Exercise is the leading predictor in Costa Rica, England, and the U.S. Smoking is the second strongest predictor in both England and the U.S. and ranks third in Costa Rica. These two variables are the only environmental predictors where ΔAUC> = .01 in at least two countries.

### Adding Other Predictors Available Only for a Subset of Datasets

In the second stage, we consider 32 additional variables available for only some datasets. Fig 3 displays the predictors that result in ΔAUC > = 0.01. The graph shows that the external health assessments are powerful predictors. Peak expiratory flow (PEF), timed chair stands, and gait speed yield ΔAUC > = 0.01 among the countries in which they are available. Grip strength is a strong contributor in Costa Rica and Taiwan.

One variable that is available only for Taiwan performs particularly well: interviewer-assessed health status (IAH, an overall health assessment in which interviewers rate respondents' health using the same five categories as SAH) is second only to grip strength in predicting mortality (net of age and sex). Notably, the interviewer’s assessment of the respondent’s health status discriminates much better (ΔAUC = 0.033) than the respondent’s own self-rating (ΔAUC = 0.023). In contrast, a rating of the respondent’s health status by a physician ranks much lower (#35 out of 57, ΔAUC = 0.005).

Several biomarkers perform very well among all countries for which they are available: serum albumin; homocysteine; and several inflammatory markers (fibrinogen, s-ICAM-1 and sE-selectin).

The entire analysis was repeated on the full samples of interviewed respondents, whether they provided biomarkers or not. Not surprisingly, these datasets have a much higher frequency of missing values, which were imputed according to the same multiple imputation procedure described in S2 Appendix. The results for Costa Rica and the U.S. are very similar to those presented here. In England, the most notable differences are that the measures of cognitive function and grip strength are stronger predictors, while CRP is a weaker predictor in the full sample than in the restricted sample. Among the Taiwanese, two of the biggest changes involve education (which becomes a stronger predictor) and history of diabetes (which becomes a weaker predictor) when models are re-estimated for all interviewed respondents.

## Discussion

To the best of our knowledge, this is the first multinational study to evaluate a wide spectrum of mortality predictors. The findings are consistent for four countries with similar levels of life expectancy (between 78.8 and 80.6 years in 2010) [12, 13], but with different cultural traditions, levels of economic development, and epidemiologic transitions over recent decades. Although only future work can establish the reproducibility of our results, the consistency of findings across countries is very encouraging, particularly in light of increasing concern about the inaccuracy and exaggeration of published results in science and medicine [14–16].

Among the variables available in all datasets, three self-reported measures of health are among the strongest predictors in all countries: IADL limitations, mobility limitations, and the simple question on overall health status (SAH). These findings, based on the AUC, are robust to two alternative measures of discrimination (S1–S4 Figs).

CRP and other inflammatory markers are notable mortality predictors, which supports the insight from Glare and colleagues that “novel prognostic factors,” including CRP and cytokines, may prove useful in recalibrating physicians’ survival predictions [17]. Two other biomarkers—serum albumin and homocysteine—are important predictors in the two countries for which they are available.

Results from Taiwan suggest that the interviewer’s rating is a better mortality predictor than the rating of overall health provided by the respondent. Interviewers may base their evaluations not only on information directly obtained in the survey, but also on their observations of the respondent’s appearance, responsiveness, cognition, and disposition [18]. Although the physicians’ ratings are weak predictors, these physicians were seeing respondents for the first time and basing their judgments on only a routine physical examination and a medical history completed by the respondent. Our findings suggest that inexpensive, easily obtained health evaluations by external observers—including lay people, caregivers, and medical personnel—may enhance prediction.

The dominance of health measures in the set of leading predictors is not altogether surprising: researchers would anticipate that measures of health—being more proximate to death—should yield stronger predictions than behaviors and social and demographic factors. Nevertheless, exercise frequency appears on the top 10 lists for three countries, sometimes above measures of disease prevalence and biomarkers. This finding raises an important caveat, namely that our analysis cannot be used to infer causal linkages between the predictors and survival. For example, exercise may discriminate well partly because of reverse causality (poor health leads to reduced exercise). However, such endogeneity is not a shortcoming when our goal is to identify warning signals (i.e., prognosis) rather than potentially modifiable risk factors.

In recognition of the importance of integrating prognosis into clinical decisions, Yourman and colleagues [19] identified 16 validated non-disease-specific prognostic indexes that forecast mortality risk for older adults over a period between six months and five years into the future (available at *eprognosis*.*ucsf*.*edu*). Concerns have been raised regarding bias, quality and generalizability of these instruments [19]. Our findings reveal additional limitations: (1) only one prognostic index incorporates SAH, despite its simplicity and predictive ability; (2) the indexes typically include several IADL or mobility limitations, but rarely the full battery typically collected in household surveys; (3) the indexes often include physicians’ assessments, rather than patients’ reports, of functional limitations, even though physicians vastly underreport functional limitations [20]; (4) no index explicitly includes information obtained from lay persons (e.g., relatives or interviewers); and (5) no index incorporates the biomarkers that we identified as strong predictors (i.e., CRP and other inflammatory markers, serum albumin, and homocysteine).

We recognize that the leading predictors of survival are likely to differ between samples of seriously ill patients—a prime concern in the clinical literature—and general population-based samples of older adults analyzed by epidemiologists and social scientists. For example, indexes on *eprognosis*.*ucsf*.*edu* intended for patients in medical facilities encompass a shorter time frame of prediction and more clinical variables (e.g., symptoms of disease) than those for community residents. These differences highlight the need to develop instruments for the appropriate population and time frame. Still, non-clinical variables (e.g., social contact and support, economic status, perceptions) are likely to predict survival among all individuals, seriously ill or not, underscoring the need for self-reported information in all settings [1].

A major strength of this analysis is that we bridge the division between clinicians and researchers interested in mortality prediction and employ a more diverse set of predictors than previous studies. Our findings are based on large nationally representative samples that span age ranges capturing the vast majority of deaths and are robust across countries and measures of discrimination. Future work is needed to determine the generalizability of our findings to younger ages and to assess variation in the leading predictors by other demographic characteristics, most notably sex. The objectives of this analysis have not required model building but this is an important next step, especially for applications that require explicit forecasts. In particular, given potential correlations between predictors and the desire for concise instruments, future research is needed to incorporate these strong predictors into parsimonious indexes that are validated with external data.

Identification of the most powerful predictors of survival is an important undertaking for social scientists, epidemiologists and clinicians. Death is a commonly used, and well-measured, endpoint in studies of environmental influences on health. Such studies place increasing emphasis on understanding the behavioral and biological pathways that underlie social stratification in health and survival and the impact of stressful experience. From a clinical perspective, long periods of illness—which characterize high life expectancy populations—underscore the need to identify individuals at high risk of death. Prognoses made by physicians and other health professionals for seriously ill patients have generally been overly optimistic [17, 21, 22]. Given advances in medical technology, growing acknowledgment of iatrogenic consequences of treatment, and a desire for cost-effective health care, optimal decision-making requires accurate prognosis of survival. Better prognostic tools may encourage physicians to have conversations with their patients about their survival prospects. Despite the inherent difficulties, such discussions may empower individuals to have more control over their final years of life and reduce unnecessary treatment that may compromise end-of-life quality.

## Supporting Information

S1 Appendix Mortality Follow-up. (DOCX)

S2 Appendix Missing Data and Multiple Imputation. (DOCX)

S3 Appendix Calculating Measures of Discrimination. (DOCX)

S1 Fig Top Ten Predictors (out of 25 variables available in all four datasets) of Five-Year Mortality After Adjustment for Age and Sex, Ranked by the Gain in AUC, NRI(>0), and IDI, Costa Rica. (DOCX)

S2 Fig Top Ten Predictors (out of 25 variables available in all four datasets) of Five-Year Mortality After Adjustment for Age and Sex, Ranked by the Gain in AUC, NRI(>0), and IDI, England. (DOCX)

S3 Fig Top Ten Predictors (out of 25 variables available in all four datasets) of Five-Year Mortality After Adjustment for Age and Sex, Ranked by the Gain in AUC, NRI(>0), and IDI, Taiwan. (DOCX)

S4 Fig Top Ten Predictors (out of 25 variables available in all four datasets) of Five-Year Mortality After Adjustment for Age and Sex, Ranked by the Gain in AUC, NRI(>0), and IDI, U.S. (DOCX)

S1 Table Information regarding sampling design and response rates for each dataset. (DOCX)

S2 Table Predictors Included in the Analysis. (DOCX)

S3 Table Variables included in the social integration index for each dataset. (DOCX)

S4 Table Items used to construct measures of ADL, IADL, and mobility limitations. (DOCX)

S5 Table Descriptive statistics for all analysis variables. (DOCX)

S6 Table Predictors with non-proportional hazards. (DOCX)

S7 Table Hazard Ratios (HR) and Odds Ratios (OR) for the Top Ten Predictors (out of 25 variables available in all four datasets) of Five-Year All-Cause Mortality After Adjusting for Age and Sex, by Country. (DOCX)

## References

[1] Christakis NA. Death foretold: prophecy and prognosis in medical care. Chicago: University of Chicago Press; 1999.

[2] Pantell M, Rehkopf D, Jutte D, Syme SL, Balmes J, Adler N. Social isolation: a predictor of mortality comparable to traditional clinical risk factors. Am J Public Health. 2013 11;103(11): 2056–62. 10.2105/AJPH.2013.301261 24028260 PMC3871270

[3] Hurd MD, McFadden D, Merrill A. Predictors of mortality among the elderly In: Wise DA, editor. Themes in the economics of aging. Chicago, IL: University of Chicago Press; 2001 pp.171–98.

[4] Belloc NB. Relationship of health practices and mortality. Prev Med. 1973 3;2(1): 67–81. 4279401 10.1016/0091-7435(73)90009-1

[5] Crimmins E, Kim JK, Vasunilashorn S. Biodemography: new approaches to understanding trends and differences in population health and mortality. Demography. 2010;47 Suppl: S41–64. 21302421 10.1353/dem.2010.0005 PMC5870619

[6] Weinstein M, Vaupel JW, Wachter KW, editors. Biosocial surveys. Washington, DC: National Academies Press; 2007. 21977539

[7] Schafer JL. Multiple imputation: a primer. Stat Methods Med Res. 1999 3;8(1): 3–15. 10347857 10.1177/096228029900800102

[8] Rubin DB. Multiple imputation after 18+ years (with discussion). J Am Stat Assoc. 1996;91: 473–89.

[9] Winship C, Radbill L. Sampling weights and regression analysis. Sociological Methods and Research. 1994;23(2): 230–57.

[10] Pencina MJ, D'Agostino RB Sr., D'Agostino RB Jr., Vasan RS. Evaluating the added predictive ability of a new marker: from area under the ROC curve to reclassification and beyond. Stat Med. 2008 1 30;27(2): 157,72; discussion 207–12. 17569110 10.1002/sim.2929

[11] Pencina MJ, D'Agostino RB S, Steyerberg EW. Extensions of net reclassification improvement calculations to measure usefulness of new biomarkers. Stat Med. 2011 1 15;30(1): 11–21. 10.1002/sim.4085 21204120 PMC3341973

[12] University of California, Berkeley (USA), Max Planck Institute for Demographic Research (Germany). Human Mortality Database. Available: www.mortality.org.

[13] The World Bank. Life expectancy at birth, total (years). Available: http://data.worldbank.org/indicator/SP.DYN.LE00.IN.

[14] Ioannidis JP, Panagiotou OA. Comparison of effect sizes associated with biomarkers reported in highly cited individual articles and in subsequent meta-analyses. JAMA. 2011 6 1;305(21): 2200–10. 10.1001/jama.2011.713 21632484

[15] Ioannidis JP. Why most published research findings are false. PLoS Med. 2005 8;2(8): E124 16060722 10.1371/journal.pmed.0020124 PMC1182327

[16] Open Science Collaboration. Estimating the reproducibility of psychological science. Science. 2015 8 28;349(6251): aac4716 10.1126/science.aac4716 26315443

[17] Glare P, Virik K, Jones M, Hudson M, Eychmuller S, Simes J, et al A systematic review of physicians' survival predictions in terminally ill cancer patients. BMJ. 2003 7 26;327(7408): 195–8. 12881260 10.1136/bmj.327.7408.195 PMC166124

[18] Todd M, Goldman N. Do interviewer health ratings predict mortality better than self-rated health? Epidemiology. 2013;24: 913–20. 24045721 10.1097/EDE.0b013e3182a713a8 PMC3968811

[19] Yourman LC, Lee SJ, Schonberg MA, Widera EW, Smith AK. Prognostic indices for older adults: a systematic review. JAMA. 2012 1 11;307(2): 182–92. 10.1001/jama.2011.1966 22235089 PMC3792853

[20] Calkins DR, Rubenstein LV, Cleary PD, Davies AR, Jette AM, Fink A, et al Failure of physicians to recognize functional disability in ambulatory patients. Ann Intern Med. 1991 3 15;114(6): 451–4. 1825267 10.7326/0003-4819-114-6-451

[21] Parkes CM. Accuracy of predictions of survival in later stages of cancer. Br Med J. 1972 4 1;2(5804): 29–31. 4111472 10.1136/bmj.2.5804.29 PMC1789062

[22] Christakis NA, Lamont EB. Extent and determinants of error in doctors' prognoses in terminally ill patients: prospective cohort study. BMJ. 2000 2 19;320(7233): 469–72. 10678857 10.1136/bmj.320.7233.469 PMC27288

