# Supplementary material for: What Matters Most for Predicting Survival? A Multinational Population-Based Cohort Study
Source: PLoS One. 2016 Jul 19;11(7):e0159273. doi: 10.1371/journal.pone.0159273 (PMC4951106; doi:10.1371/journal.pone.0159273)
Supplement: S1 Appendix — (DOCX) [file pone.0159273.s001.docx]

# S1 Appendix. Mortality Follow-up

"For NHANES, vital status as of January 1, 2012 was determined by linkage with death certificate records from the National Death Index. For SEBAS, survival status as of January 1, 2012 was ascertained through linkage with the death certificate file maintained by the Department of Health and the household registration database maintained by the Ministry of the Interior. For CRELES, survival status as of March 31, 2014 was established in two ways: (1) through the computer records in the National Death Registry, and (2) during the second (2006-2008) and third (2008-2010) waves of home visits. The computer follow-up used the unique identification number (the *cédula*) assigned to Costa Ricans. For the foreigners in the sample (approx. 3%), survival was established only in the field because they did not have a unique identification number with which to link them to the Registry." (Glei et al. 2016, Supporting Information, S1 Appendix, p. 28)[1]

For ELSA, vital status in February 2012 was determined based on the National Health Service Central Register.

# References

1. Glei DA, Goldman N, Risques RA, Rehkopf DH, Dow WH, Rosero-Bixby L, et al. Predicting survival: telomere length versus conventional predictors. PLOS ONE. 2016. doi: 10.1371/journal.pone.0152486
