# Supplementary material for: What Matters Most for Predicting Survival? A Multinational Population-Based Cohort Study
Source: PLoS One. 2016 Jul 19;11(7):e0159273. doi: 10.1371/journal.pone.0159273 (PMC4951106; doi:10.1371/journal.pone.0159273)
Supplement: S2 Appendix — (DOCX) [file pone.0159273.s002.docx]

# S2 Appendix. Missing Data and Multiple Imputation

The individual variables with a large proportion (>5%) of missing data comprised: depressive symptoms (25% in CRELES; 7% in NHANES); instrumental activities of daily living (IADL) limitations (14% in NHANES); self-reported mobility (14% in CRELES, 6% in NHANES); blood pressure (12% in ELSA, 11% in NHANES); BMI (8% in CRELES); physician-assessed health status (7% in SEBAS); assets (6% in SEBAS); waist circumference (6% in NHANES); waist-hip ratio (6% in CRELES); and blood-based markers (5-7% in ELSA). We used multiple imputation to create five imputed datasets. The prediction equations included all of the variables in this analysis plus two auxiliary variables that were correlated with non-response or with predictors that had extensive missing data: whether the respondent was interviewed by proxy (not applicable for ELSA or NHANES) and alcohol consumption.

"Then, we estimated the model for each imputed dataset and combined the five sets of estimates using Rubin’s rules [1]. All measures of fit and predictive ability were calculated for each dataset and then averaged following the same rules." (Glei et al., 2016, Supporting Information, S1 Appendix, p. 29)[2]

Despite the widespread use of multiple imputation for handling missing data, the method is based on several assumptions, most notable of which is the “missing at random” assumption. This assumption means that, in the presence of controls for observed variables, the probability that a particular variable X has missing data does not depend on the value of X. Although frequently violated, this assumption cannot be tested. See Allison [3] for further details regarding the assumptions underlying multiple imputation.

# References

1. Royston P, Carlin JB, White IR. Multiple imputation of missing values: new features for mim. Stata Journal. 2009;9(2): 252-64.

2. Glei DA, Goldman N, Risques RA, Rehkopf DH, Dow WH, Rosero-Bixby L, et al. Predicting survival: telomere length versus conventional predictors. PLOS ONE. 2016. doi: 10.1371/journal.pone.0152486

3. Allison PD. Multiple imputation for missing data:  a cautionary tale. Sociological Methods and Research. 2000;28(3): 301-9.
