# Supplementary material for: What Matters Most for Predicting Survival? A Multinational Population-Based Cohort Study
Source: PLoS One. 2016 Jul 19;11(7):e0159273. doi: 10.1371/journal.pone.0159273 (PMC4951106; doi:10.1371/journal.pone.0159273)
Supplement: S3 Appendix — (DOCX) [file pone.0159273.s003.docx]

# S3 Appendix. Calculating Measures of Discrimination

## Predicted Probability of Dying Within Five Years Post-Exam

We used a Cox model with non-proportional hazards, where the hazard at age $t$ for an individual with covariates $x$ takes the form:

$\boldsymbol{\lambda}\left( \boldsymbol{t}\boldsymbol{,x} \right)\boldsymbol{=}\boldsymbol{\lambda}_{\boldsymbol{0}}\left( \boldsymbol{t} \right)\boldsymbol{e}^{\boldsymbol{x}^{\boldsymbol{'}}\boldsymbol{\beta}\boldsymbol{(t)}}$ ,

where $\lambda_{0}\left( t \right)$ is the baseline hazard and $\beta(t)$ is a vector of time-varying covariates. The model was estimated by maximizing Cox’s partial likelihood with the Efron correction for ties using the “stcox” command in Stata 12.1. We used the “tvc()” option to specify covariates that vary with respect to time (i.e., age).

To estimate the baseline survival function we used a custom program written by Germán Rodríguez that extends the procedures in Stata to time-varying covariates. This program computes the hazard contribution at each failure time by solving equation 4.34 in Kalbfleisch and Prentice (p.115) [1], using a direct solution if there are no ties and a Newton iterative procedure in the event of ties. The probability of surviving five years after the exam is then calculated as a product of conditional survival probabilities for all ages between the age at exam and five years later.

## Measures of Discrimination: AUC, NRI(>0), and IDI

We used the “roctab” procedure in Stata 12.1 to compute the AUC by comparing the model-based predicted probability of dying within five years post-exam (see above) with the observed outcome (death vs. survival).

"The traditional Net Reclassification Improvement (NRI) requires clinically meaningful risk strata, but we use a newer category-free version, NRI(>0), which quantifies the correct movement of model-based probabilities when additional markers are added to the model: upward for decedents and downward for survivors [2]. An alternative measure, the Integrated Discrimination Improvement (IDI), can be interpreted as the difference in discrimination slopes of models with and without the new markers [3], where the discrimination slope is the absolute difference in the average prediction between those who experienced the event and those who did not [4]" (Goldman et al. 2015, pp. 902-903)[5].

“Although there are no established benchmarks, Pencina and colleagues suggest ΔAUC>0.01represents a meaningful improvement, while NRI(>0) greater than 0.6 indicates a strong contribution and NRI(>0) between 0.2 and 0.6 implies moderate improvement [3, 6]. Researchers do not provide a corresponding gauge for IDI” (Goldman et al., 2015, pp. 903) [5]. We show these benchmarks for ΔAUC and NRI (>0) in S1-S4 Figs.

The NRI(>0) and IDI were computed using a custom program in Stata written by Germán Rodríguez.

# References

1. Kalbfleisch JD, Prentice RL. The statistical analysis of failure time data. 2nd edition ed. New York: Wiley, 2002.

2. Pencina MJ, D'Agostino RB S, Steyerberg EW. Extensions of net reclassification improvement calculations to measure usefulness of new biomarkers. Stat Med 2011 Jan 15;30(1):11-21.

3. Pencina MJ, D'Agostino RB,Sr., D'Agostino RB,Jr., Vasan RS. Evaluating the added predictive ability of a new marker: from area under the ROC curve to reclassification and beyond. Stat Med 2008 Jan 30;27(2):157,72; discussion 207-12.

4. Yates JF. External correspondence:  decomposition of the mean probability score. Organizational Behavior and Human Performance 1982;30:132-56.

5. Goldman N, Glei DA. Quantifying the value of biomarkers for predicting mortality. Ann Epidemiol 2015 Dec;25(12):901,906.e4.

6. Pencina MJ, D'Agostino RB, Pencina KM, Janssens ACW, Greenland P. Interpreting incremental value of markers added to risk prediction models. Am J Epidemiol 2012 Sep 15;176(6):473-81.
