# Supplementary material for: What Matters Most for Predicting Survival? A Multinational Population-Based Cohort Study
Source: PLoS One. 2016 Jul 19;11(7):e0159273. doi: 10.1371/journal.pone.0159273 (PMC4951106; doi:10.1371/journal.pone.0159273)
Supplement: S2 Fig — (DOCX) [file pone.0159273.s005.docx]

**S2 Fig. Top Ten Predictors (out of 25 variables available in all four datasets) of Five-Year Mortality After Adjustment for Age and Sex, Ranked by the Gain in AUC, NRI(>0), and IDI, England**

Abbreviations: ADL, Activities of daily living; CRP, C-reactive protein; Educ, Educational attainment; IADL, Instrumental Activities of Daily Living; SAH, Self-assessed health status, Social Int., Social integration.

Weak

Moderate

Strong

Improvement

Meaningful

Improvement
