# Supplementary material for: What Matters Most for Predicting Survival? A Multinational Population-Based Cohort Study
Source: PLoS One. 2016 Jul 19;11(7):e0159273. doi: 10.1371/journal.pone.0159273 (PMC4951106; doi:10.1371/journal.pone.0159273)
Supplement: S1 Table — (DOCX) [file pone.0159273.s008.docx]

**S1 Table. Information regarding sampling design and response rates for each dataset**

|  | **CRELES, Wave 1** | **ELSA, Wave 2** | **SEBAS 2006** | **NHANES, Wave 2005-06** |
| --- | --- | --- | --- | --- |
| Population sampled | Costa Rica, residents | England, persons living in private households | Taiwan, excluding mountainous areas populated largely by aboriginals | U.S., civilian, noninstitutionalized persons |
| Age range | 60+ | 52+ | 53+ | All ages |
| Sampling design | Multi-stage sample drawn from the 2000 census database [1] | The ELSA cohort was sampled from participants in the 1998, 1999, and 2001 HSE [2]. Respondents who completed the ELSA Wave 1 interview (*n*=11,391; response rate 67%) were eligible for Wave 2. | The SEBAS cohort comprises a random subsample of participants in the TLSA, which was drawn using multistage probability sampling [3]. The youngest group (aged 53-60) was first interviewed in the 2003 TLSA (*n*=1,599; response rate 79%). The older group (aged 60+) was drawn from those interviewed in the 1999 TLSA (*n*=4,440; response rate 90%); those who completed the 2000 SEBAS exam (*n*=1023; 63% participation) were eligible for the 2006 SEBAS. | Stratified, multistage probability sample [4] |
| Subgroup(s) oversampled | Persons aged 95+ |  | Persons aged 77+;  Urban residents | Low-income persons;  Adolescents 12-19 years;  Persons aged 65+;  African Americans;  Mexican Americans |
| Dates of fieldwork | November 2004-  September 2006 | June 2004-  July 2005 | August 2006-  January 2007 | January 2005-  December 2006 |
| Interview completed: | | | | |
| N | 2,827 | 8,780 | 1,284 | 10,348 |
| Response Rate | 70% | 82% | 87% | 80% |
| Site of physical exam | Home | Home | Hospital | Mobile examination center |
| Participated in exam: | | | | |
| N | 2,738 | 7,666 | 1,036 | 9,950 |
| % of those interviewed | 97% | 87% | 81% | 96% |
| Excluded from analysis because respondent did not provide a blood sample: | | | | |
| N | 44 | 1,438 | 4 | 1,526 |
| Excluded from analysis because vital status at end of follow-up was unknown: | | | | |
| N | 0 | 115**^a^** | 0 | 0 |
| **Analysis sample** | **2,694** | **6,113** | **1,032** | **2,023 (aged 50+)^b^** |

Abbreviations: CRELES, Costa Rican Study on Longevity and Healthy Aging; ELSA, English Longitudinal Study of Aging; HSE, Health Survey for England; NHANES, National Health and Nutrition Examination Survey; SEBAS, Social Environment and Biomarkers of Aging Study; TLSA, Taiwan Longitudinal Study of Aging.

^a^ For ELSA, those with unknown vital status are included for the purposes of multiple imputation, but then deleted for the main analyses.

^b^ In order to be comparable with the other datasets, we restricted the analysis sample for NHANES to respondents aged 50 and older.

# References

1. Rosero-Bixby L, Fernández X, Dow WH. CRELES: Costa Rican Longevity and Health Aging Study, 2005 (Costa Rica Estudio de Longevidad y Envejecimiento Saludable): Sampling and Methods. Ann Arbor, MI: Inter-university Consortium for Political and Social Research; 2010

2. Steptoe A, Breeze E, Banks J, Nazroo J. Cohort profile: the English Longitudinal Study of Ageing. Int J Epidemiol. 2013 Dec;42(6):1640-8.

3. Chang M, Lin H, Chuang Y, Goldman N, Peterson CE, Glei DA, et al. Social Environment and Biomarkers of Aging Study (SEBAS) in Taiwan, 2000 and 2006: main documentation for SEBAS longitudinal public use data (released 2012). Ann Arbor, MI: Inter-university Consortium for Political and Social Research [distributor]; 2012

4. Curtin LR, Mohadjer LK, Dohrmann SM, Montaquila JM, Kruszon-Moran D, Mirel LB, et al. The National Health and Nutrition Examination Survey:  sample design, 1999-2006.  National Center for Health Statistics.  Vital Health Stat. Hyattsville, Maryland: National Center for Health Statistics, Centers for Disease Control and Prevention, U.S. Department of Health and Human Services; 2012
