# Supplementary material for: What Matters Most for Predicting Survival? A Multinational Population-Based Cohort Study
Source: PLoS One. 2016 Jul 19;11(7):e0159273. doi: 10.1371/journal.pone.0159273 (PMC4951106; doi:10.1371/journal.pone.0159273)
Supplement: S2 Table — (DOCX) [file pone.0159273.s009.docx]

**S2 Table. Predictors Included in the Analysis**

|  | Costa Rica [CRELES] | **England [ELSA]** | Taiwan [SEBAS] | **U.S. [NHANES]** |
| --- | --- | --- | --- | --- |
| **Environmental Predictors** |  |  |  |  |
| **Demographic characteristics** |  |  |  |  |
| 1. Age at exam | 60+, mean=70.4, SD=8.1 | 52+, mean=65.9, SD=9.9 | 53+, mean=66.2, SD=8.9 | 50+, mean=64.0, SD=10.6 |
| 1. Sex | Female: *n*=1461 (52%) | Female: *n*=3320 (53%) | Female: *n*=477 (46%) | Female: *n*=989 (54%) |
| 1. Race/ethnicity | N/A | White  non-white | Mainlander  Hakka  Fukienese/other | Non-Hispanic white  Non-Hispanic black  Hispanic  Other/mixed race |
| 1. Marital status^a^ | ✓ | ✓ | ✓ | ✓ |
| **Socioeconomic status** |  |  |  |  |
| 1. Education^b^ | Completed years | Categorical | Completed years | Categorical |
| Low | 0-2 years | No qualifications | 0-5 years | < HS diploma/GED |
| Medium low | 3-5 years | Foreign or other qualification | 6 years | HS diploma/GED |
| Medium high | 6 years | NVQ1/CSE/NVQ2/  GCE O level | 7-11 years | Some college or associate degree |
| High | 7+ years | NVQ3/GCE A level/higher | 12+ years | College graduate or higher |
| 1. Annual Income | Respondent & spouse | Respondent & spouse | Respondent & spouse  [from 2003 TLSA] | Household, Categorical^c^ |
| 1. Assets | Index (0-10)^d^ | Respondent & spouse | Respondent & spouse | N/A |
| **Psychosocial factors** |  |  |  |  |
| 1. Social integration index^e^ | Based on 5 items  Cronbach’s α=0.61 | Based on 8 items  Cronbach’s α=0.79 | Based on 10 items  Cronbach’s α=0.68 | Based on 3 items  Cronbach’s α=0.73 |
| 1. Socially isolated^f^ | ✓ | ✓ | ✓ | ✓ |
| 1. Perceived stress index [1] | N/A | N/A | Based on 10 items  Cronbach’s α=0.82 | N/A |
| 1. Church attendance^g^ | How often do you attend religious services?  [Some categories combined] | N/A | How often do you go to church or worship in temples?  [from 2003 TLSA] | How often do you attend church or religious services per year?  [recoded into categories] |
| Never | Never |  | Never | 0 |
| Rarely | Occasionally |  | Rarely | 1-10 times/year |
| Sometimes | Monthly/Biweekly |  | Sometimes | 10-51 times/year |
| Often | Weekly/Daily |  | Often | 52+ times/year |
| 1. Religious beliefs | N/A | N/A | Index based on 4 items  [from 2003 TLSA]  Cronbach’s α=0.86 | N/A |
| **Health-related behaviors** |  |  |  |  |
| 1. Smoking status^h^ | ✓ | ✓ | ✓ | ✓ |
| 1. Exercise frequency^g^ | Dummy | Frequency of moderate and vigorous exercise  [recoded] | Categorical | Frequency of moderate and vigorous leisure-time physical activities in past 30 days, [recoded into categories] |
| None | < 3 times/week,  past 12 months | Never/hardly ever | None | None |
| Low |  | Moderate <= weekly & no vigorous | <3 times/week | <12 times/30 days |
| Medium | 3+ times/week,  past 12 month | Moderate > weekly & no vigorous | 3-5 times/week | 12-29 but Vigorous<12 |
| High |  | Vigorous <= weekly | 6+ times/week | 30+ but Vigorous<12 |
| Very high |  | Vigorous > weekly |  | Vigorous 12+ times/30 days |
| **Underlying health** |  |  |  |  |
| **Biomarkers** |  |  |  |  |
| 1. Systolic blood pressure | 2^nd^ reading taken in home | Mean of 2^nd^ and 3^rd^ readings taken in home | Mean of 2^nd^ and 3^rd^  readings taken in home | Mean of 2^nd^ and 3^rd^ readings taken in MEC |
| 1. Diastolic blood pressure | 2^nd^ reading taken in home | Mean of 2^nd^ and 3^rd^ readings taken in home | Mean of 2^nd^ and 3^rd^  readings taken in home | Mean of 2^nd^ and 3^rd^ readings taken in MEC |
| 1. Resting pulse | N/A | Measured in home | Measured in home | Measured in MEC |
| 1. Total cholesterol (TC) | ✓ | ✓ | ✓ | ✓ |
| 1. HDL cholesterol | ✓ | ✓ | ✓ | ✓ |
| 1. Ratio of TC/HDL | ✓ | ✓ | ✓ | ✓ |
| 1. Triglycerides | ✓ | ✓ | ✓ | N/A^i^ |
| 1. HbA1c | ✓ | ✓ | ✓ | ✓ |
| 1. Fasting glucose | ✓ | ✓ | ✓ | N/A^i^ |
| 1. Body mass index | ✓ | ✓ | ✓ | ✓ |
| 1. Waist circumference | ✓ | ✓ | ✓ | ✓ |
| 1. Waist/hip ratio | ✓ | ✓ | ✓ | N/A |
| 1. Interleukin-6 | N/A | N/A | ✓ | N/A |
| 1. C-reactive protein | ✓ | ✓ | ✓ | ✓ |
| 1. Fibrinogen | N/A | ✓ | ✓ | N/A |
| 1. sICAM-1 | N/A | N/A | ✓ | N/A |
| 1. sE-selectin | N/A | N/A | ✓ | N/A |
| 1. WBC count | N/A | N/A | ✓ | ✓ |
| 1. DHEAS | ✓ | N/A | ✓ | N/A |
| 1. Urinary cortisol^j^ | N/A^i^ | N/A | ✓ | N/A |
| 1. Urinary epinephrine^j^ | N/A^i^ | N/A | ✓ | N/A |
| 1. Urinary norepinephrine^j^ | N/A^i^ | N/A | ✓ | N/A |
| 1. Serum creatinine | ✓ | N/A | ✓ | ✓ |
| 1. Homocysteine | N/A | N/A | ✓ | ✓ |
| 1. Serum albumin | N/A | N/A | ✓ | ✓ |
| 1. IGF-1 | N/A | N/A | ✓ | N/A |
| **Self-reported measures of health** |  |  |  |  |
| 1. Self-assessed health status^k^ | ✓ | ✓ | ✓ | ✓ |
| 1. Number of ADL limitations | Based on 5 ADLs | Based on 6 ADLs | Based on 6 ADLs | Based on 5 ADLs |
| 1. Number of IADL limitations | Based on 4 IADLs | Based on 7 IADLs | Based on 6 IADLs | Based on 5 IADLs |
| 1. Number of mobility limitations | Based on 4 tasks | Based on 10 tasks | Based on 9 tasks | Based on 8 tasks |
| 1. History of diabetes | Dr. ever told you that you have had diabetes (high levels of blood sugar)? | Dr. ever told you have diabetes or high blood sugar? | Dr. ever diagnosed… | Ever told by a Dr. or other health professional that you have diabetes or sugar diabetes? |
| 1. History of cancer | Dr. ever told you that you have cancer or a  malignant tumor, not including small skin tumors? | Dr. ever told you have cancer or a malignant tumor (excluding minor skin cancers)? | Dr. ever diagnosed cancer or a malignant tumor? | Ever told by a Dr. or other health professional that you have cancer or a malignancy of any kind? |
| 1. History of stroke | Dr. ever told you that you have had a stroke? | Dr. ever told you have had a stroke (cerebrovascular disease)? | Dr. ever diagnosed stroke (cerebral hemorrhage)? | Dr. or other health professional ever told you that you had a stroke? |
| 1. History of heart disease | Dr. ever told you that you have had a heart attack or heart disease without having a heart attack? | Dr. ever told you that you have had angina, a heart attack (including myocardial infarction or coronary thrombosis), or congestive heart failure? | Dr. ever diagnosed heart disease (not including palpitations)? | Dr. or other health professional ever told you that you had congestive heart failure, coronary heart disease, angina (also called angina pectoris), or a heart attack (also called a myocardial infarction)? |
| 1. Hospital stays, past 12 months | N/A | N/A | ✓ | ✓ |
| 1. Hospital days, past 12 months | ✓ | N/A | ✓ | N/A |
| 1. 5+ medications, % | Prescription meds | N/A | All meds | Prescription meds |
| 1. Depressive symptoms | 15 items from Yesavage’s Geriatric Depression Scale  Cronbach’s α=0.85 | 8 abbreviated items  (i.e., binary response) from CES-D  Cronbach’s α=0.80 | 10-item subset of CES-D  Cronbach’s α=0.83 | PHQ-9 Depression Index  Cronbach’s α=0.83 |
| **Health assessments** |  |  |  |  |
| 1. Interviewer-assessed health status^l^ | N/A | N/A | ✓ | N/A |
| 1. Physician-assessed health status^l^ | N/A | N/A | ✓ | N/A |
| 1. Cognitive function | Based on tasks from the MMSE [2] (basic orientation, immediate & delayed recall, follow a 3-stage command, copy a geometric design) as well as a reverse recall task | Based on various tasks  (time orientation,  immediate & delayed recall, prospective memory, word-finding, letter cancellation) | Based on several items from the SPMSQ [3] (basic orientation, serial subtraction), a word recall task from the modified RAVLT [4], & a modified version of the Digits Backwards Test [5] | N/A |
| 1. Grip strength | Max from 3 trials on dominant hand | Max from 3 trials on each hand | Max from 3 trials on each hand | N/A |
| 1. Peak expiratory flow (PEF) | Max from 3 trials | Max from 3 trials | Max from 3 trials | N/A |
| 1. Timed walk | From a sitting position, rise from chair and walk 3m at normal pace,  1 trial | N/A^i^ | From a standing position, walk 3m at normal pace, Max from 2 trials | N/A |
| 1. Timed chair stands | 5 complete stands,  adjusted for chair height | 5 complete stands | 5 complete stands, adjusted for chair height | N/A |

Abbreviations: ADL, Activities of daily living; DHEAS, Dehydroepiandrosterone sulfate; GED, General equivalency diploma; HDL, High-density lipoprotein cholesterol; HS, High school; IADL, Instrumental activities of daily living; IGF-1, Insulin-like growth factor 1; HbA1c, Glycosylated hemoglobin; MEC, Mobil Examination Center; N/A, Not Available; sICAM‑1, Soluble intercellular adhesion molecule 1; sE-selectin, Soluble E-selectin; WBC, White blood cell.

Note: Variables that were available in all four datasets are highlighted in green.

^a^ Coded to the following categories: Married/Partner; Widowed; Divorced/Separated; and Never Married.

^b^ Because education was coded as categorical in ELSA and NHANES, we recoded education (completed years) into categories for CRELES and SEBAS as well. Given differences across countries in the distribution of education, we attempted (to the extent possible) to categorize education into quartiles within each sample.

^c^ In NHANES, household income was reported in categories (<US$5,000; 5,000-9,999; 10,000-14,999;…65,000-74,999; $75,000+; <$20,000; >$20,000). We recoded income to the mid-point of each category and coded the open-ended categories as follows: $75,000+=$85,000; >$20,000=$40,000.

^d^ One point is assigned for each of the following: 1) exterior walls, floor, and roof of the domicile are all in good condition; 2) separate room for cooking; 3) fuel used most for cooking is electric or gas; 4) potable water in the house; 5) indoor toilet; 6) refrigerator; 7) television; 8) phone; 9) washing machine; 10) car.

^e^ See Table S3 for a detailed list of the components included in the social integration index for each country. Because the level of measurement varies across items, we standardize each of the components (based on the within-country distribution) and then calculate the mean across valid items if at least 75% items are valid.

^f^ Dichotomous variable indicating those falling in the bottom decile on the social integration index (based on the within-country distribution) in order to capture the most socially isolated individuals.

^g^ Response categories were recoded in order to obtain more comparable measures across datasets.

^h^ Coded to the following categories: never smoked; former smoker; current smoker.

^i^ Not included in the analysis because it was measured only for a subset of respondents.

^j^ Standardized by urinary creatinine.

^k^ Based on a simple question that is typically worded “How would you rate your current state of health?” and has five response categories ranging from “poor” to “excellent.”

^l^ The interviewer and the physician were each asked independently to assess the respondent’s current state of health using a question similar to the one asked of the respondent (see above).

# References

1. Cohen S, Kamarck T, Mermelstein R. A global measure of perceived stress. J Health Soc Behav. 1983;24(4):385-96.

2. Folstein, M.,Susan Folstein and Paul McHugh. "Mini-Mental State": A Practical Method for Grading the Cognitive State of Patients for the Clinician. J.Psychiat.Res. 1975;12:189-98.

3. Pfeiffer E. A short portable mental status questionnaire for the assessment of organic brain deficit in elderly patients. J Am Geriatr Soc. 1975 Oct;23(10):433-41.

4. Lezak MD. Neuropsychological Assessment, Second edition. New York: Oxford University Press, 1983.

5. Wechsler D. WAIS-R Manual. New York: Psychological Corporation, 1981.
