# Supplementary material for: What Matters Most for Predicting Survival? A Multinational Population-Based Cohort Study
Source: PLoS One. 2016 Jul 19;11(7):e0159273. doi: 10.1371/journal.pone.0159273 (PMC4951106; doi:10.1371/journal.pone.0159273)
Supplement: S3 Table — (DOCX) [file pone.0159273.s010.docx]

**S3 Table. Variables included in the social integration index for each dataset**

| **Variable** | **Definition and Coding** | CRELES | ELSA | SEBAS | NHANES |
| --- | --- | --- | --- | --- | --- |
| Network size | ELSA: Number of friends/relatives with whom respondent has a close relationship; recoded 0, 1-2, 3-4, 5-6, 7-8, 9-11, 12+  SEBAS: Number of friends/relatives the respondent lives with or has regular contact; recoded <5, 5-7, 8-10, 11-14, 15-19, 20-29, 30+ | N/A | ✓ | ✓ | N/A |
| Network range | Number of types of relationships in social network; One point each for spouse/partner, kids, other relatives, non-relatives; range=0-4 | N/A | ✓ | ✓ | N/A |
| Frequency of interaction  with network members | ELSA: We calculate the average for two questions (how often meet up with: family, friends); response categories from 0=never to 5=three or more times per week  SEBAS: How often the respondent has contact with friends, neighbors, or relatives; response categories from 0=never to 4=nearly daily | N/A | ✓ | ✓ | N/A |
| Household size | Top-coded at 7+ | ✓ | ✓ | ✓ | ✓ |
| Does not live alone | Dummy indicating that the respondent does not live alone | ✓ | ✓ | ✓ | ✓ |
| Married/partner | Dummy indicating that the respondent is married or lives with a companion | ✓ | ✓ | ✓ | ✓ |
| Number of friends | ELSA: Number of close friends; recoded 0, 1, 2, 3, 4, 5+  NHANES: Number of close friends; recoded 0, 1-2, 3-4, 5-9, 10-19, 20+  SEBAS: Number of close friends and neighbors with whom the respondent has weekly contact; recoded 0, 1-2, 3-4, 5-9, 10-19, 20+ | N/A | ✓ | ✓ | ^a^ |
| Number of children | Top-coded at 8+ | ✓ | ^a^ | ^a^ | N/A |
| Number of grandchildren | CRELES: recoded 0, 1-4, 5-9, 10-14,15-24, 25+  SEBAS: recoded 0, 1-2, 3-5, 6-9, 10-14, 15+ | ✓ | ^a^ | ^a^ | N/A |
| Religious attendance | How often the respondent attends religious services; Response categories from 0=never to 3=often (SEBAS); to 4=more than once a week (CRELES) | ^a^ | N/A | ✓ | ^a^ |
| Participation in  social organizations | CRELES: Summed number of hours per week spent in church and civic activities and then categorized into 0, 1, 2, 3-4, 5+ hours per week  ELSA: Number of committee meetings per year; recoded 0, 1, 2, 3-4, 5-6, 7-12, 13+  SEBAS: One point for each of 8 social organizations/activities in which the respondent participates | ^a^ | ✓ | ✓ | N/A |
| Volunteer work | Dummy indicating that the respondent does volunteer work. | N/A | N/A | ✓ | N/A |

^a^ Item was dropped from the index because the item-rest correlation (i.e., the correlation between that item and the scale formed by all the remaining items) was less than 0.
