# Supplementary material for: What Matters Most for Predicting Survival? A Multinational Population-Based Cohort Study
Source: PLoS One. 2016 Jul 19;11(7):e0159273. doi: 10.1371/journal.pone.0159273 (PMC4951106; doi:10.1371/journal.pone.0159273)
Supplement: S4 Table — (DOCX) [file pone.0159273.s011.docx]

**S4 Table. Items used to construct measures of ADL, IADL, and mobility limitations**

|  | Costa Rica [CRELES] | **England [ELSA]** | Taiwan [SEBAS] | **U.S. [NHANES]** |
| --- | --- | --- | --- | --- |
| **ADL limitations** | Based on 5 ADLs | Based on 6 ADLs | Based on 6 ADLs | Based on 5 ADLs |
| Bathing | ✓ | ✓ | ✓ |  |
| Dressing |  | ✓ | ✓ | ✓ |
| Eating | ✓ | ✓ | ✓ | ✓ |
| Getting in or out of bed | ✓ | ✓ | ✓ | ✓ |
| Standing up from/sitting in a chair |  |  |  | ✓ |
| Moving around the house | ✓ | ✓ | ✓ | ✓ |
| Using the toilet | ✓ | ✓ | ✓ |  |
|  |  |  |  |  |
| **IADL limitations** | Based on 4 IADLs | Based on 7 IADLs | Based on 6 IADLs | Based on 5 IADLs |
| Buy personal items | ✓ | ✓ | ✓ |  |
| Manage money | ✓ | ✓ | ✓ | ✓ |
| Take bus/train alone |  |  | ✓ |  |
| Physical work at home |  | ✓ | ✓ |  |
| Light chores at home |  |  | ✓ | ✓ |
| Make phone call |  | ✓ | ✓ |  |
| Preparing meals | ✓ | ✓ |  | ✓ |
| Going out (e.g., shopping) | ✓ |  |  | ✓ |
| Going to social events |  |  |  | ✓ |
| Taking medications |  | ✓ |  |  |
| Using a map |  | ✓ |  |  |
|  |  |  |  |  |
| **Mobility limitations** | Based on 4 tasks | Based on 10 tasks | Based on 9 tasks | Based on 8 tasks |
| Lift/Carry |  | ✓ | ✓ | ✓ |
| Climb 2+ flights of stairs | ✓ | ✓ | ✓ |  |
| Climb 1 flight of stairs |  | ✓ |  |  |
| Climb 10 steps |  |  |  | ✓ |
| Bend/kneel/stoop/squat |  | ✓ | ✓ | ✓ |
| Walk > 1 mile |  |  |  |  |
| Walk several blocks/200-300m | ✓ |  | ✓ |  |
| Walk one block/100 yards |  | ✓ |  |  |
| Walk ¼ mile |  |  |  | ✓ |
| Stand for 15 min |  |  | ✓ |  |
| Stand for 2 hours/long period |  |  | ✓ | ✓ |
| Raise arms overhead | ✓ | ✓ | ✓ | ✓ |
| Grasp with fingers |  |  | ✓ | ✓ |
| Pick up a small coin from a table |  | ✓ |  |  |
| Run a short distance |  |  | ✓ |  |
| Push/pull a large object | ✓ | ✓ |  |  |
| Get up from a chair after sitting for a long period |  | ✓ |  |  |
| Sitting for long period |  | ✓ |  | ✓ |

Abbreviations: ADL, Activities of daily living; IADL, Instrumental activities of daily living.

# References

1. Cohen S, Kamarck T, Mermelstein R. A global measure of perceived stress. J Health Soc Behav. 1983;24(4):385-96.

2. Folstein, M.,Susan Folstein and Paul McHugh. "Mini-Mental State": A Practical Method for Grading the Cognitive State of Patients for the Clinician. J.Psychiat.Res. 1975;12:189-98.

3. Pfeiffer E. A short portable mental status questionnaire for the assessment of organic brain deficit in elderly patients. J Am Geriatr Soc. 1975 Oct;23(10):433-41.

4. Lezak MD. Neuropsychological Assessment, Second edition. New York: Oxford University Press, 1983.

5. Wechsler D. WAIS-R Manual. New York: Psychological Corporation, 1981.
