# Supplementary material for: What Matters Most for Predicting Survival? A Multinational Population-Based Cohort Study
Source: PLoS One. 2016 Jul 19;11(7):e0159273. doi: 10.1371/journal.pone.0159273 (PMC4951106; doi:10.1371/journal.pone.0159273)
Supplement: S5 Table — (DOCX) [file pone.0159273.s012.docx]

**S5 Table. Descriptive statistics for all analysis variables^a^**

|  | Costa Rica [CRELES] (*n*=2694) | |  | **England [ELSA]**  **(*n*=6113)** | |  | Taiwan [SEBAS]  (*n*=1032) | |  | U.S. [NHANES]  (N=2023) | |
| --- | --- | --- | --- | --- | --- | --- | --- | --- | --- | --- | --- |
|  | Mean (SD)  or *n* (%) | Range |  | Mean (SD)  or *n* (%) | Range |  | Mean (SD)  or *n* (%) | Range |  | Mean (SD)  or *n* (%) | Range |
| Died within 5 years, *n* (%) | 694 (16.3) |  |  | 522 (8.9) |  |  | 112 (10.2) |  |  | 279 (9.5) |  |
|  |  |  |  |  |  |  |  |  |  |  |  |
| **Environmental** |  |  |  |  |  |  |  |  |  |  |  |
| **Demographic** |  |  |  |  |  |  |  |  |  |  |  |
| Age at exam, mean (SD) | 70.4 (8.1) | 60-109 |  | 65.9 (9.9) | 52-93 |  | 66.0 (9.2) | 53-98 |  | 64.0 (10.6) | 50-90^b^ |
| Female, *n* (%) | 1461 (52.3) |  |  | 3320 (53.0) |  |  | 477 (46.0) |  |  | 989 (53.8) |  |
| Non-white, *n* (%) | N/A |  |  | 87 (1.9) |  |  | N/A |  |  | N/A |  |
| Non-hispanic white, *n* (%) | N/A |  |  | N/A |  |  | N/A |  |  | 1200 (80.5) |  |
| Non-hispanic black, *n* (%) | N/A |  |  | N/A |  |  | N/A |  |  | 441 (9.7) |  |
| Hispanic, *n* (%) | N/A |  |  | N/A |  |  | N/A |  |  | 327 (6.0) |  |
| Other/mixed race, *n* (%) | N/A |  |  | N/A |  |  | N/A |  |  | 55 (3.8) |  |
| Fukienese/other, *n* (%) | N/A |  |  | N/A |  |  | 736 (73.9) |  |  | N/A |  |
| Hakka, *n* (%) | N/A |  |  | N/A |  |  | 160 (16.2) |  |  | N/A |  |
| Mainlander, *n* (%) | N/A |  |  | N/A |  |  | 131 (10.0) |  |  | N/A |  |
| Marital status |  |  |  |  |  |  |  |  |  |  |  |
| Married, *n* (%) | 1347 (60.8) |  |  | 4336 (71.3) |  |  | 783 (77.0) |  |  | 1192 (65.0) |  |
| Widowed, *n* (%) | 869 (21.4) |  |  | 1005 (16.3) |  |  | 200 (17.5) |  |  | 393 (14.5) |  |
| Divorced/separated, *n* (%) | 256 (10.5) |  |  | 506 (7.9) |  |  | 28 (2.5) |  |  | 336 (16.4) |  |
| Never married, *n* (%) | 222 (7.4) |  |  | 266 (4.6) |  |  | 21 (2.0) |  |  | 102 (4.1) |  |
|  |  |  |  |  |  |  |  |  |  |  |  |
| **Socioeconomic** |  |  |  |  |  |  |  |  |  |  |  |
| Education^c^ |  |  |  |  |  |  |  |  |  |  |  |
| Low, *n* (%) | 1010 (27.6) |  |  | 2176 (37.9) |  |  | 265 (27.3) |  |  | 638 (19.4) |  |
| Medium low, *n* (%) | 871 (23.7) |  |  | 553 (8.9) |  |  | 383 (39.3) |  |  | 515 (27.9) |  |
| Medium high, *n* (%) | 465 (27.7) |  |  | 1396 (22.5) |  |  | 129 (11.3) |  |  | 491 (27.9) |  |
| High, *n* (%) | 348 (21.0) |  |  | 1988 (30.7) |  |  | 255 (22.1) |  |  | 379 (24.8) |  |
| Annual Income^d^, mean (SD) | 3.9 (7.5) | 0-126 |  | 37.1 (31.3) | 0-572 |  | 15.4 (24.6) | 0-406 |  | 48.4 (26.8) | 3-85 |
| Assets^e^, mean (SD) | 8.2 (1.7) | 0-10 |  | 104.2 (252.5) | -131 to 4326 |  | 76.0 (155.6) | 0-1892 |  | N/A |  |
|  |  |  |  |  |  |  |  |  |  |  |  |
| **Psychosocial** |  |  |  |  |  |  |  |  |  |  |  |
| Social integration, mean (SD) | 0.0 (0.6) | -1.9 to 1.2 |  | 0.1 (0.6) | -1.6 to 1.8 |  | 0.2 (0.5) | -1.5 to 1.6 |  | 0.04 (0.8) | -1.7 to 1.6 |
| Perceived stress, mean (SD) | N/A |  |  | N/A |  |  | 10.0 (6.6) | 0-34 |  | N/A |  |
| Church attendance |  |  |  | -- |  |  |  |  |  |  |  |
| Never, *n* (%) | 590 (16.5) |  |  |  |  |  | 219 (19.1) |  |  | 652 (34.8) |  |
| Rarely, *n* (%) | 701 (25.5) |  |  |  |  |  | 225 (22.6) |  |  | 243 (12.6) |  |
| Sometimes, *n* (%) | 201 (7.9) |  |  |  |  |  | 429 (42.6) |  |  | 310 (15.2) |  |
| Often, *n* (%) | 1202 (50.2) |  |  |  |  |  | 159 (15.7) |  |  | 818 (37.4) |  |
| Religious beliefs, mean (SD) | N/A |  |  | N/A |  |  | 1.4 (0.9) | 0-3 |  | N/A |  |
|  |  |  |  |  |  |  |  |  |  |  |  |
| **Health behaviors** |  |  |  |  |  |  |  |  |  |  |  |
| Smoking status |  |  |  |  |  |  |  |  |  |  |  |
| Never, *n* (%) | 1528 (56.6) |  |  | 2259 (36.6) |  |  | 640 (61.9) |  |  | 937 (47.3) |  |
| Former, *n* (%) | 952 (33.4) |  |  | 2981 (48.4) |  |  | 196 (18.5) |  |  | 731 (34.9) |  |
| Current, *n* (%) | 214 (10.0) |  |  | 873 (15.0) |  |  | 196 (19.6) |  |  | 355 (17.8) |  |
| Exercise frequency |  |  |  |  |  |  |  |  |  |  |  |
| None, *n* (%) | 2048 (68.6) |  |  | 827 (14.1) |  |  | 547 (54.2) |  |  | 950 (39.7) |  |
| Low, *n* (%) |  |  |  | 922 (15.2) |  |  | 81 (7.4) |  |  | 307 (17.3) |  |
| Medium, *n* (%) | 646 (31.4) |  |  | 1852 (30.3) |  |  | 131 (12.0) |  |  | 234 (14.3) |  |
| High, *n* (%) |  |  |  | 1317 (21.2) |  |  | 273 (26.4) |  |  | 318 (15.6) |  |
| Very high, *n* (%) |  |  |  | 1195 (19.3) |  |  |  |  |  | 214 (13.1) |  |
|  |  |  |  |  |  |  |  |  |  |  |  |
| **Underlying Health** |  |  |  |  |  |  |  |  |  |  |  |
| **Biomarkers** |  |  |  |  |  |  |  |  |  |  |  |
| SBP (mmHg), mean (SD) | 142.6 (23.5) | 73-233 |  | 135.3 (18.8) | 80-225 |  | 139.1 (20.8) | 91-222 |  | 130.0 (20.2) | 71-232 |
| DBP (mmHg), mean (SD) | 83.1 (12.6) | 40-130 |  | 75.4 (11.1) | 31-128 |  | 79.1 (12.0) | 47-135 |  | 70.3 (13.2) | 0-118 |
| Pulse (beats/min), mean (SD) | N/A |  |  | 67.7 (11.4) | 34-149 |  | 73.8 (11.0) | 48-117 |  | 70.3 (11.4) | 40-124 |
| TC (mmol/L), mean (SD) | 5.6 (1.3) | 1.5-12.6 |  | 5.9 (1.2) | 2.0-12.3 |  | 5.2 (1.0) | 2.2-10.2 |  | 5.3 (1.1) | 2.1-11.9 |
| HDL (mmol/L), mean (SD) | 1.2 (0.4) | 0.2-3.0 |  | 1.5 (0.4) | 0.5-4.1 |  | 1.2 (0.4) | 0.4-3.3 |  | 1.5 (0.4) | 0.4-4.9 |
| Ratio of TC/HDL, mean (SD) | 5.0 (1.5) | 1.4-33.2 |  | 4.1 (1.0) | 1.6-11.3 |  | 4.4 (1.2) | 1.8-9.9 |  | 3.9 (1.2) | 1.3-9.2 |
| Triglycerides (mmol/L), mean (SD) | 1.9 (1.1) | 0.3-11.0 |  | 1.8 (1.2) | 0.4-18.7 |  | 1.3 (0.8) | 0.2-7.4 |  | N/A |  |
| HbA1c (proportion), mean (SD) | 0.06 (0.01) | 0.02-0.14 |  | 0.06 (0.01) | 0.03-0.14 |  | 0.06 (0.01) | 0.041-0.145 |  | 0.06 (0.01) | 0.04-0.14 |
| Glucose (mmol/L), mean (SD) | 6.0 (2.2) | 1.0-27.8 |  | -- |  |  | 6.0 (1.9) | 3.4-21.0 |  | N/A |  |
| BMI, mean (SD) | 26.9 (5.3) | 8.2-81.7 |  | 27.8 (4.8) | 14.9-56.2 |  | 24.8 (3.5) | 16.1-39.1 |  | 29.0 (6.4) | 13.4-59.1 |
| Waist (cm), mean (SD) | 93.8 (12.6) | 24-169 |  | 95.6 (13.0) | 61-172 |  | 83.8 (9.8) | 45-125 |  | 100.8 (15.6) | 55-159 |
| Waist/hip ratio, mean (SD) | 0.9 (0.1) | 0.3-1.6 |  | 0.9 | 0.6-1.3 |  | 0.9 (0.1) | 0.5-1.1 |  | N/A |  |
| IL-6 (pg/mL), mean (SD) | N/A |  |  | N/A |  |  | 4.1 (8.9) | 0.1-154.4 |  | N/A |  |
| CRP (nmol/L), mean (SD) | 56.3 (67.4) | 28.4-1400.0 |  | 40.0 (85.4) | 1.9-2000.0 |  | 25.4 (69.8) | 0.1-1142.0 |  | 43.9 (69.7) | 1.0-841.9 |
| Fibrinogen (g/dL), mean (SD) | N/A |  |  | 3.2 (0.7) | 1.0-8.9 |  | 3.3 (0.7) | 0.5-6.5 |  | N/A |  |
| sICAM-1(ng/mL), mean (SD) | N/A |  |  | N/A |  |  | 270.5 (98.4) | 3-969 |  | N/A |  |
| sE-selectin (ng/mL), mean (SD) | N/A |  |  | N/A |  |  | 43.4 (34.4) | 4.4-363.2 |  | N/A |  |
| WBC count (10^9^/L), mean (SD) | N/A |  |  | N/A |  |  | 0.006 (0.002) | 0.002-0.015 |  | 0.007 (0.003) | 0.002-0.056 |
| DHEAS (μmol/L), mean (SD) | 1.5 (1.2) | 0.3-13.0 |  | N/A |  |  | 2.9 (2.0) | 0.02-18.2 |  | N/A |  |
| Cortisol (μmol/mol creatinine), mean (SD) | N/A |  |  | N/A |  |  | 6.5 (10.2) | 0.2-192.4 |  | N/A |  |
| Epinephrine (μmol/mol creatinine), mean (SD) | N/A |  |  | N/A |  |  | 2.5 (1.6) | 0.3-15.9 |  | N/A |  |
| Norepinephrine (μmol/mol creatinine), mean (SD) | N/A |  |  | N/A |  |  | 17.8 (9.2) | 1.9-84.5 |  | N/A |  |
| SCr (μmol/L), mean (SD) | 95.5 (32.1) | 7.1-592.3 |  | N/A |  |  | 84.9 (38.4) | 35.4-671.8 |  | 88.3 (41.5) | 35.4-1573.5 |
| Hcy (μmol/L), mean (SD) | N/A |  |  | N/A |  |  | 11.9 (6.5) | 0.5-124.5 |  | 9.6 (3.9) | 3.6-125 |
| Serum albumin (g/dL), mean (SD) | N/A |  |  | N/A |  |  | 44.0 (3.0) | 28-53 |  | 42.0 (3.2) | 23-52 |
| IGF-1 (μg/L), mean (SD) | N/A |  |  | N/A |  |  | 148.8 (63.2) | 11-524 |  | N/A |  |
|  |  |  |  |  |  |  |  |  |  |  |  |
| **Self-reported health** |  |  |  |  |  |  |  |  |  |  |  |
| SAH, mean (SD) | 2.7 (1.0) | 1-5 |  | 3.3 (1.1) | 1-5 |  | 3.1 (1.0) | 1-5 |  | 3.3 (1.1) | 1-5 |
| ADL limitations, mean (SD) | 0.3 (1.0) | 0-5 |  | 0.3 (0.9) | 0-6 |  | 0.2 (0.9) | 0-6 |  | 0.5 (1.1) | 0-5 |
| IADL limitations, mean (SD) | 0.6 (1.2) | 0-4 |  | 0.4 (0.9) | 0-7 |  | 0.7 (1.4) | 0-6 |  | 0.6 (1.3) | 0-5 |
| Mobility limitations, mean (SD) | 1.3 (1.3) | 0-4 |  | 1.9 (2.5) | 0-10 |  | 1.8 (2.4) | 0-9 |  | 1.7 (2.3) | 0-8 |
| Hx of diabetes, *n* (%) | 524 (21.1) |  |  | 359 (5.9) |  |  | 167 (16.7) |  |  | 363 (13.9) |  |
| Hx of cancer, *n* (%) | 180 (5.8) |  |  | 440 (7.0) |  |  | 46 (4.2) |  |  | 313 (14.9) |  |
| Hx of stroke, *n* (%) | 147 (3.8) |  |  | 141 (2.3) |  |  | 46 (4.1) |  |  | 157 (5.8) |  |
| Hx of heart disease, *n* (%) | 471 (15.3) |  |  | 519 (8.5) |  |  | 178 (16.1) |  |  | 353 (13.9) |  |
| Hospital stays, mean (SD) | N/A |  |  | N/A |  |  | 0.2 (0.7) | 0-11 |  | 0.2 (0.6) | 0-6 |
| Hospital days, mean (SD) | 1.1 (7.3) | 0-200 |  | N/A |  |  | 1.8 (8.7) | 0-186 |  | N/A |  |
| 5+ medications, *n* (%) | 738 (26.2) |  |  | N/A |  |  | 277 (25.4) |  |  | 593 (26.6) |  |
| Depressive symptoms, mean (SD) | 18.5 (21.2) | 0-99 |  | 1.5 (1.9) | 0-8 |  | 4.8 (5.6) | 0-30 |  | 2.6 (3.7) | 0-27 |
|  |  |  |  |  |  |  |  |  |  |  |  |
| **Health assessments** |  |  |  |  |  |  |  |  |  |  |  |
| IAH, mean (SD) | N/A |  |  | N/A |  |  | 3.8 (1.0) | 1-5 |  | N/A |  |
| PAH, mean (SD) | N/A |  |  | N/A |  |  | 2.3 (0.8) | 1-4 |  | N/A |  |
| Cognitive function, mean (SD) | 88.5 (10.5) | 4-100 |  | 0.0 (0.6) | -2.9 to 2.6 |  | 16.5 (3.7) | 0-23 |  | N/A |  |
| Unable to do grip strength test, *n* (%) | 152 (3.1) |  |  | 38 (0.6) |  |  | 33 (2.7) |  |  | N/A |  |
| Grip strength (kg), mean (SD) | 26.8 (8.9) | 4-58 |  | 32.1 (11.7) | 1-75 |  | 28.0 (10.4) | 2-68 |  | N/A |  |
| Unable to do PEF test, *n* (%) | 406 (9.5) |  |  | 374 (6.3) |  |  | 28 (2.7) |  |  | N/A |  |
| PEF (L/min), mean (SD) | 306.7 (113.9) | 35-730 |  | 375.9 (140.4) | 28-948 |  | 335.2 (136.6) | 26-800 |  | N/A |  |
| Unable to do timed walk, *n* (%) | 462 (9.4) |  |  | N/A |  |  | 37 (3.3) |  |  | N/A |  |
| Walking speed (m/sec), mean (SD) | 0.6 (0.2) | 0.1-2.0 |  | N/A |  |  | 0.9 (0.3) | 0.1-2.1 |  | N/A |  |
| Unable to do chair stands test, *n* (%) | 626 (12.9) |  |  | 651 (11.1) |  |  | 88 (7.9) |  |  | N/A |  |
| Chair stand speed (stands/sec), mean (SD) | 0.4 (0.1) | 0.1-1.7 |  | 0.5 (0.1) | 0.1-1.4 |  | 0.5 (0.2) | 0.1-1.9 |  | N/A |  |

Abbreviations: ADL, Activities of daily living; BMI, Body mass index; CRP, C-reactive protein; DBP, Diastolic blood pressure; DHEAS, Dehydroepiandrosterone sulfate; HbA1c, Glycosylated hemoglobin; HDL, High-density lipoprotein cholesterol; Hcy, Homocysteine; IADL, Instrumental Activities of Daily Living; IAH, Interviewer-assessed health status; IGF-1, Insulin-like growth factor 1; IL-6, Interleukin-6; N/A, Not Available; PAH, Physician-assessed health status; PEF, Peak expiratory flow; SAH, Self-assessed health status; SCr, Serum creatinine; sICAM-1, Soluble intercellular adhesion molecule 1; sE-selectin, Soluble E-selectin; TC, Total cholesterol; WBC, White blood cell.

^a^ Data represent unweighted numbers (*n*) and weighted analyses for all other statistics (i.e., %, mean, SD).

^b^ In order to protect confidentiality, respondents aged 85 and older were top-coded in the NHANES public-use dataset. We have coded age for these respondents to the sex-specific mean age (88.7 for men, 89.5 for women) among the U.S. population aged 85 and older on 1/1/2006 based on population estimates from the Human Mortality Database [1].

^c^ Given that educational attainment varied across the four countries, we specified the categories based on the within-country distribution (see Table S2 for details).

^d^ Expressed in thousands of U.S. dollars using 2005 exchange rates: US$1=₡477.79 Costa Rica colón= £0.5493 British pound=NT$31.71 (Taiwan) (<http://www.exchangerate.com/country-information/>). Values represent the reported household income for the U.S. and the income for the respondent and his/her spouse in the other three populations. Because income was reported in categories in the U.S., the maximum is not a true maximum.

^e^ In Costa Rica, assets are measured by an index (see Table S2 for details). In England and Taiwan, assets represent the financial wealth of the respondent and his/her spouse; those values have been converted to thousands of US dollars using the same exchange rates noted above.

# References

1. University of California, Berkeley (USA), Max Planck Institute for Demographic Research (Germany). Human Mortality Database. [www.mortality.org](http://www.mortality.org) (accessed March 1, 2016).
