# Supplementary material for: What Matters Most for Predicting Survival? A Multinational Population-Based Cohort Study
Source: PLoS One. 2016 Jul 19;11(7):e0159273. doi: 10.1371/journal.pone.0159273 (PMC4951106; doi:10.1371/journal.pone.0159273)
Supplement: S6 Table — (DOCX) [file pone.0159273.s013.docx]

**S6 Table. Predictors with non-proportional hazards**

| Predictor | **Costa Rica**  **[CRELES]** | **England**  **[ELSA]** | **Taiwan**  **[SEBAS]** | **U.S.**  **[NHANES]** |
| --- | --- | --- | --- | --- |
| **Demographic characteristics** |  |  |  |  |
| Marital status |  | 🡫 |  |  |
| **Socioeconomic status** |  |  |  |  |
| Education |  |  | 🡫 | 🡫 |
| Income |  |  |  | 🡫 |
| Assets |  | 🡩 |  |  |
| **Psychosocial factors** |  |  |  |  |
| Social integration index |  |  | 🡫 |  |
| **Biomarkers** |  |  |  |  |
| Systolic blood pressure (SBP) | 🡫🡩(77.7) |  |  |  |
| Diastolic blood pressure (DBP) | 🡫 |  | 🡫 |  |
| Resting pulse |  | 🡫 |  | 🡫 |
| Total cholesterol |  |  | 🡫 |  |
| HDL cholesterol |  |  |  |  |
| Triglycerides | 🡫 |  |  |  |
| Fasting glucose | 🡫 |  |  |  |
| Body mass index (BMI) | 🡫 |  |  |  |
| Waist circumference | 🡫 |  |  |  |
| Waist/hip ratio | 🡫 |  |  |  |
| C-reactive protein |  |  |  | 🡫 |
| DHEAS |  |  | 🡫 |  |
| Serum creatinine (SCr) | 🡫 |  |  |  |
| Serum albumin |  |  | 🡫 |  |
| **Self-reported measures of health** |  |  |  |  |
| Number of ADL limitations | 🡫 |  |  |  |
| Number of IADL limitations | 🡫 |  |  |  |
| History of diabetes | 🡫 |  |  |  |
| History of cancer |  | 🡫 |  | 🡫 |
| History of stroke | 🡫 |  |  | 🡫 |
| History of heart disease | 🡫 |  |  |  |
| 5+ medications | 🡫 |  |  |  |
| **Health assessments** |  |  |  |  |
| Unable to do timed walk | 🡫 |  |  |  |
| Unable to do chair stands | 🡫 |  |  |  |

🡫 Effect decreases with age

🡩 Effect increases with age

🡫🡩(*t*) Positive effect (i.e., higher SBP is associated with higher mortality) that diminishes with age until age *t*, then reverses sign (i.e., higher SBP is associated with lower mortality) and the magnitude intensifies with age thereafter.
