# Supplementary material for: What Matters Most for Predicting Survival? A Multinational Population-Based Cohort Study
Source: PLoS One. 2016 Jul 19;11(7):e0159273. doi: 10.1371/journal.pone.0159273 (PMC4951106; doi:10.1371/journal.pone.0159273)
Supplement: S7 Table — (DOCX) [file pone.0159273.s014.docx]

**S7 Table. Hazard Ratios (HR) and Odds Ratios (OR) for the Top Ten Predictors (out of 25 variables available in all four datasets) of Five-Year All-Cause Mortality After Adjusting for Age and Sex, by Country**

|  | **CRELES**  **(Costa Rica, N=2694)** | | |  | **ELSA**  **(England, N=6113)** | | |  | **SEBAS**  **(Taiwan, N=1032)** | | |  | **NHANES**  **(U.S., N=2023)** | | |
| --- | --- | --- | --- | --- | --- | --- | --- | --- | --- | --- | --- | --- | --- | --- | --- |
|  | **Rank** | **HR** | **95% C.I.** |  | **Rank** | **OR** | **95% C.I.** |  | **Rank** | **HR** | **95% C.I.** |  | **Rank** | **HR** | **95% C.I.** |
| Number of IADL limitations^a^ | 1 | 2.33^b^ | 1.86-2.93 |  | 4 | 1.37 | 1.28-1.48 |  | 1 | 1.61 | 1.39-1.85 |  | 2 | 1.50 | 1.37-1.65 |
| Number of Mobility limitations^a^ | 2 | 1.66 | 1.52-1.81 |  | 3 | 1.46 | 1.34-1.60 |  | 2 | 1.67 | 1.40-2.00 |  | 1 | 1.60 | 1.41-1.80 |
| Self-Assessed Health (SAH) | 4 |  |  |  | 1 |  |  |  | 3 |  |  |  | 3 |  |  |
| Poor (ref) |  | 1.00 |  |  |  | 1.00 |  |  |  | 1.00 |  |  |  | 1.00 |  |
| Fair |  | 0.65 | 0.51-0.83 |  |  | 0.40 | 0.29-0.56 |  |  | 0.64 | 0.33-1.23 |  |  | 0.69 | 0.47-1.03 |
| Good |  | 0.48 | 0.37-0.62 |  |  | 0.25 | 0.18-0.35 |  |  | 0.32 | 0.16-0.62 |  |  | 0.45 | 0.31-0.67 |
| Very Good |  | 0.45 | 0.32-0.62 |  |  | 0.16 | 0.11-0.23 |  |  | 0.31 | 0.15-0.66 |  |  | 0.37 | 0.24-0.56 |
| Excellent |  | 0.56 | 0.40-0.79 |  |  | 0.14 | 0.09-0.22 |  |  | 0.17 | 0.05-0.53 |  |  | 0.24 | 0.13-0.44 |
| Number of ADL limitations^a^ | 3 | 1.95^b^ | 1.63-2.34 |  | 8 | 1.29 | 1.20-1.39 |  | 9 | 1.21 | 1.09-1.33 |  | 4 | 1.38 | 1.26-1.51 |
| C-Reactive Protein^a^ | 10 | 1.13 | 1.08-1.19 |  | 5 | 1.30 | 1.20-1.41 |  | 6 | 1.19 | 1.08-1.30 |  | 5 | 1.53 | 1.24-1.89 |
| Exercise frequency | 5 |  |  |  | 2 |  |  |  |  |  |  |  | 6 |  |  |
| None (ref) |  | 1.00 |  |  |  | 1.00 |  |  |  |  |  |  |  | 1.00 |  |
| Low |  |  |  |  |  | 0.47 | 0.35-0.63 |  |  |  |  |  |  | 0.52 | 0.34-0.79 |
| Medium |  | 0.50 | 0.39-0.64 |  |  | 0.38 | 0.29-0.49 |  |  |  |  |  |  | 0.70 | 0.45-1.09 |
| High |  |  |  |  |  | 0.28 | 0.20-0.39 |  |  |  |  |  |  | 0.51 | 0.36-0.74 |
| Very high |  |  |  |  |  | 0.21 | 0.14-0.31 |  |  |  |  |  |  | 0.37 | 0.19-0.73 |
| History of diabetes | 9 | 2.74^b^ | 1.65-4.56 |  |  |  |  |  | 4 | 1.88 | 1.22-2.89 |  | 8 | 2.07 | 1.57-2.71 |
| History of heart disease | 6 | 3.20^b^ | 1.93-5.31 |  |  |  |  |  |  |  |  |  | 7 | 1.93 | 1.50-2.49 |
| History of stroke | 7 | 6.11^b^ | 2.94-12.67 |  |  |  |  |  | 7 | 2.89 | 1.63-5.11 |  |  |  |  |
| Smoking status |  |  |  |  | 6 |  |  |  |  |  |  |  | 9 |  |  |
| Never (ref) |  |  |  |  |  | 1.00 |  |  |  |  |  |  |  | 1.00 |  |
| Former |  |  |  |  |  | 1.43 | 1.13-1.81 |  |  |  |  |  |  | 1.18 | 0.90-1.56 |
| Current |  |  |  |  |  | 2.97 | 2.18-4.05 |  |  |  |  |  |  | 2.51 | 1.74-3.63 |
| Education |  |  |  |  |  |  |  |  |  |  |  |  | 10 |  |  |
| Low (ref) |  |  |  |  |  |  |  |  |  |  |  |  |  | 1.00 |  |
| Medium low |  |  |  |  |  |  |  |  |  |  |  |  |  | 0.53^b^ | 0.27-1.03 |
| Medium high |  |  |  |  |  |  |  |  |  |  |  |  |  | 0.63^b^ | 0.33-1.21 |
| High |  |  |  |  |  |  |  |  |  |  |  |  |  | 0.25^b^ | 0.10-0.61 |
| Depressive symptoms^a^ |  |  |  |  |  |  |  |  | 5 | 1.35 | 1.15-1.58 |  |  |  |  |
| History of cancer |  |  |  |  | 7 | 4.81^b^ | 2.94-7.85 |  |  |  |  |  |  |  |  |
| HbA1c^a^ | 8 | 1.17 | 1.09-1.26 |  |  |  |  |  |  |  |  |  |  |  |  |
| Social integration^a^ |  |  |  |  | 9 | 0.74 | 0.67-0.82 |  | 10 | 0.59^b^ | 0.40-0.88 |  |  |  |  |
| Diastolic blood pressure^a^ |  |  |  |  |  |  |  |  | 8 | 1.70^b^ | 1.76-2.45 |  |  |  |  |
| Marital status |  |  |  |  | 10 |  |  |  |  |  |  |  |  |  |  |
| Married/partner (ref) |  |  |  |  |  | 1.00 |  |  |  |  |  |  |  |  |  |
| Widowed |  |  |  |  |  | 1.18^b^ | 0.66-2.12 |  |  |  |  |  |  |  |  |
| Divorced/separated |  |  |  |  |  | 2.94^b^ | 1.80-4.79 |  |  |  |  |  |  |  |  |
| Never married |  |  |  |  |  | 2.18^b^ | 1.09-4.37 |  |  |  |  |  |  |  |  |

^a^ The HR/OR represents the effect per SD of the specified predictor.

^b^ The effect of the predictor varied with age; the HR/OR shown here represents the effect at age 60.
